# New record of the genus *Amblypalpus* Mitrofanov & Strunkova (Acari, Prostigmata, Tenuipalpidae) with description of a new species from Saudi Arabia

**DOI:** 10.3897/BDJ.13.e156638

**Published:** 2025-07-17

**Authors:** Nasreldeen Ahmed Elgoni, Jawwad Hassan Mirza, Fahad Jaber Alatawi

**Affiliations:** 1 Department of Plant Protection, College of Food and Agriculture Sciences, King Saud University, Riyadh, Saudi Arabia Department of Plant Protection, College of Food and Agriculture Sciences, King Saud University Riyadh Saudi Arabia

**Keywords:** diagnosis, tarsal claw, taxonomy, morphology, diversity, *Pisonia* sp.

## Abstract

**Background:**

*Amblypalpus* Mitrofanov & Strunkova (Prostigmata, Tenuipalpidae) is a small genus in the family Tenuipalpidae, representing five species reported from Iran, Tajikistan and South Africa. This study describes a new species of *Amblypalpus*, A. pisoniae **sp. nov**., reported from the south-western region (Jazan) of Saudi Arabia (SA).

**New information:**

The genus *Amblypalpus* Mitrofanov & Strunkova is reported for the first time from SA, collected from *Pisonia* sp. (Nyctaginaceae) with a new species, *A.pisoniae* **sp. nov.** The new species is described and illustrated, based on females and males. Additionally, the diagnosis of the genus *Amblypalpus* is updated.

## Introduction

The genus *Amblypalpus* Mitrofanov & Strunkova (Acari, Prostigmata, Tenuipalpidae) was erected by Mitrofanov and Strunkova (1978) with *A.narsikulovi* Mitrofanov & Strunkova as type species and comprises five species namely; *A.iraniensis* Farzan, Asadi &Ueckermann, *A.thymus* Farzan, Asadi and Ueckermann (from Iran), *A.masakii* (Ehara &Ueckermann), *A.thomissus* (Meyer) (from South Africa) and *A.narsikulovi* Mitrofanov & Strunkova (from Tajikistan) (Mitrofanov and Strunkova 1978, Meyer 1979, Mesa et al. 2009, Farzan et al. 2013).

Ehara and Ueckermann (2003) described the species *Tenuipalpusmasakii*; later, Mesa et al. (2009) transferred this species from *Tenuipalpus* to *Amblypalpus*. Recently, Farzan et al. (2013) reviewed and updated the diagnosis of two genera, *Priscapalpus* DeLeon, 1961 and *Amblypalpus* and transferred *P.thomissus* Meyer, 1979, to *Amblypalpus*. Later, Castro et al. (2017) developed the key to known species of the genus *Amblypalpus*. Farzan et al. (2013) reported in the genus *Amblypalpus* diagnosis the presence of uncinate tarsal claws and pad-like empodium. In contrast, all species of this genus have tarsal claw and empodium, pad-like. Recently, Beard and Seeman (2025) divided *Amblypalpus* into two groups: *Amblypalpus* sensu stricto, which includes only one species, *A.narsikulovi* and *Amblypalpus* sensu lato, which consists of the other four species.

In the present study, the genus *Amblypalpus* is reported for the first time from Saudi Arabia (SA) with a description of a new species, *A.pisoniae* **sp. nov.**, found on *Pisonia* sp. (Nyctaginaceae) from south-western (Jazan), SA. Additionally, the diagnosis of *Amblypalpus* has been updated.

## Materials and methods

Different wild vegetations in the southern regions of SA were comprehensively surveyed from January to July 2024 to collect mites. The mite specimens were collected by shaking the foliage parts of the plant over white sheets and preserved in small vials containing 70% ethanol. Specimens were examined under a phase-contrast microscope (DM2500, Leica, Wetzlar, Germany). The species was identified by following the published key (Castro et al. 2017). The images of different body parts were captured using an auto-montage software system (Syncroscopy, Cambridge, UK) attached to a phase contrast microscope (DM2500, Leica, Germany). These images were used as templates and illustrated by Adobe Illustrator software (Adobe Systems Inc., San Jose, CA, USA). The morphological terminology used in the new species description follows Lindquist (1985) and Mesa et al. (2009). All measurements are given in micrometres (μm). The holotype measurements for the morphological features are provided as a single value followed by those of the paratype in parentheses. The values were rounded off to the nearest integer and regarded as the length of the morphological traits unless otherwise specified, i.e. number of setae. Body dimensions measured as *v*_2_–*h*_1_ (length) and *sc*_2_–*sc*_2_ (width). The type specimens of the new species have been deposited at the King Saud University Museum of Arthropods (KSMA, Acarology section), Department of Plant Protection, College of Food and Agriculture Sciences, King Saud University, Riyadh, Saudi Arabia.

## Taxon treatments

### Amblypalpus pisoniae

Elgoni, Mirza & Alatawi sp. nov.

314405D0-118E-572C-88E1-B6ED91CAC729

7B4C92BB-6008-4498-BCD7-2E0ABBF57725

#### Materials

- **Type status:** Holotype. **Occurrence:** catalogNumber: KSMAAS24-Ten-Amb-H; recordedBy: N.A. Elgoni; individualCount: 1; sex: Female; lifeStage: adult; occurrenceID: 3D1A726B-FBAD-5A6D-AA95-30414EBF42DE; **Taxon:** scientificName: Amblypalpuspisoniae; kingdom: Animalia; phylum: Arthropoda; class: Arachnida; order: Prostigmata; family: Tenuipalpidae; genus: Amblypalpus; **Location:** country: Saudi Arabia; stateProvince: Jazan; locality: Ayban; verbatimCoordinates: 17°16.320'N 43°2.629'E; decimalLatitude: 17.272; decimalLongitude: 43.04382; georeferenceProtocol: GPS; **Identification:** identifiedBy: Nasreldeen Ahmed Elgoni; dateIdentified: 2024; **Event:** samplingProtocol: shaking plant foliage; eventDate: 25/2/2024; habitat: Pisonia spp.; **Record Level:** language: en; collectionCode: Mites; basisOfRecord: Slide Mounted Specimen
- **Type status:** Paratype. **Occurrence:** catalogNumber: KSMAAS24-Ten-Amb-P1; recordedBy: N.A. Elgoni; individualCount: 1; sex: Female; lifeStage: adult; occurrenceID: 6B18ACD0-D5E7-5FAF-99AA-48EE8F0D8697; **Taxon:** scientificName: Amblypalpuspisoniae; kingdom: Animalia; phylum: Arthropoda; class: Arachnida; order: Prostigmata; family: Tenuipalpidae; genus: Amblypalpus; **Location:** country: Saudi Arabia; stateProvince: Jazan; locality: Ayban; verbatimCoordinates: 17°16.320'N 43°2.629'E; decimalLatitude: 17.272; decimalLongitude: 43.04382; georeferenceProtocol: GPS; **Identification:** identifiedBy: Nasreldeen Ahmed Elgoni; dateIdentified: 2024; **Event:** samplingProtocol: shaking plant foliage; eventDate: 25/2/2024; habitat: Pisonia spp.; **Record Level:** language: en; collectionCode: Mites; basisOfRecord: Slide Mounted Specimen
- **Type status:** Paratype. **Occurrence:** catalogNumber: KSMAAS24-Ten-Amb-P2; recordedBy: N.A. Elgoni; individualCount: 1; sex: Male; lifeStage: adult; occurrenceID: DAB48C65-2FAE-52E8-B35B-DFF40059C487; **Taxon:** scientificName: Amblypalpuspisoniae; kingdom: Animalia; phylum: Arthropoda; class: Arachnida; order: Prostigmata; family: Tenuipalpidae; genus: Amblypalpus; **Location:** country: Saudi Arabia; stateProvince: Jazan; locality: Ayban; verbatimCoordinates: 17°16.320'N 43°2.629'E; decimalLatitude: 17.272; decimalLongitude: 43.04382; georeferenceProtocol: GPS; **Identification:** identifiedBy: Nasreldeen Ahmed Elgoni; dateIdentified: 2024; **Event:** samplingProtocol: shaking plant foliage; eventDate: 25/2/2024; habitat: Pisonia spp.; **Record Level:** language: en; collectionCode: Mites; basisOfRecord: Slide Mounted Specimen
- **Type status:** Paratype. **Occurrence:** catalogNumber: KSMAAS24-Ten-Amb-P3; recordedBy: N.A. Elgoni; individualCount: 1; sex: Male; lifeStage: adult; occurrenceID: E220106C-5802-51DE-8AD2-605116DDF738; **Taxon:** scientificName: Amblypalpuspisoniae; kingdom: Animalia; phylum: Arthropoda; class: Arachnida; order: Prostigmata; family: Tenuipalpidae; genus: Amblypalpus; **Location:** country: Saudi Arabia; stateProvince: Jazan; locality: Ayban; verbatimCoordinates: 17°16.320'N 43°2.629'E; decimalLatitude: 17.272; decimalLongitude: 43.04382; georeferenceProtocol: GPS; **Identification:** identifiedBy: Nasreldeen Ahmed Elgoni; dateIdentified: 2024; **Event:** samplingProtocol: shaking plant foliage; eventDate: 25/2/2024; habitat: Pisonia spp.; **Record Level:** language: en; collectionCode: Mites; basisOfRecord: Slide Mounted Specimen

#### Description

**Description of female (n = 2)**

**Dorsum** (Fig. 1a and b). Body oval in shape. Length of body from setae *v*_2_–*h*_1_ 157 (159); width from *sc*_2_–*sc*_2_ 85 (86). Propodosoma anteriorly deeply notched 22 (17), with two long medial lobes and without lateral lobes. Median area of propodosoma with irregular lines, lateral area with irregular longitudinal striae; first and second pairs of propodosomal setae *v*_2_ and *sc*_1_ subequal in length and longer than third pair *sc*_2_. Opisthosoma with irregular, transverse, longitudinal striae, opisthosoma with one pair of pore posterior setae *d*_3_. Lengths of setae: *v*_2_ 14 (13), *sc*_1_ 15 (18), *sc*_2_ 8 (11), *c*_1_ 15 (8), *c*_3_ 8 (7), *d*_1_ 7 (5), *d*_3_ 9 (11), *e*_1_ 11 (10), *f*_3_ 9 (10), *h*_1_ 7(8), *h*_2_ 9 (8). Distances between setae: *v*_2_–*v*_2_ 28 (24), *sc*_1_–*sc*_1_ 54 (57), *c*_1_–*c*_1_ 27 (26), *c*_3_–*c*_3_ 95 (103), *d*_1_–*d*_1_ 5 (8), *d*_3_–*d*_3_ 82 (88), *e*_1_–*e*_1_ 6 (8), *f*_3_–*f*_3_ 55 (56), *h*_1_–*h*_1_ 14 (12), *h*_2_–*h*_2_ 34 (30). All dorsal setae lanceolate serrate.

**Venter** (Fig. 2a-d). Ventral cuticle with transverse striae medially, longitudinal striae laterally; genital and ventral region fused into single plate with transverse striae; lateral area with longitudinal striae; three pairs of intercoxal setae (1*a*, 3*a*, 4*a*), with 1*a* long and 4*a* longer than 3*a.* Coxa I with 3 setae (1*b*_1_, 1*b*_2_, 1*c*); two pairs of genital setae (*g*_1–2_) present; two pairs of anal setae (*ps*_2–3_) present. Spermatheca (Fig. 2d). Spermathecal duct long, narrow and with two bulbs. Lengths of setae: 1*a* 44 (41), 1*b*_1_ 14 (15), 1*b*_2_ 33 (30), 1*c* 16 (13), 2*b* 32 (31), 2*c* 14 (13), 3*a* 16 (16), 3*b* 18 (18), 4*a* 33 (34), 4*b* 19 (16), *ag* 13 (14), *g*_1_ 10 (11), *g*_2_ 11 (12), *ps*_2_ 12 (13), *ps*_3_ 12 (11). All ventral setae smooth.

**Figure 3.**
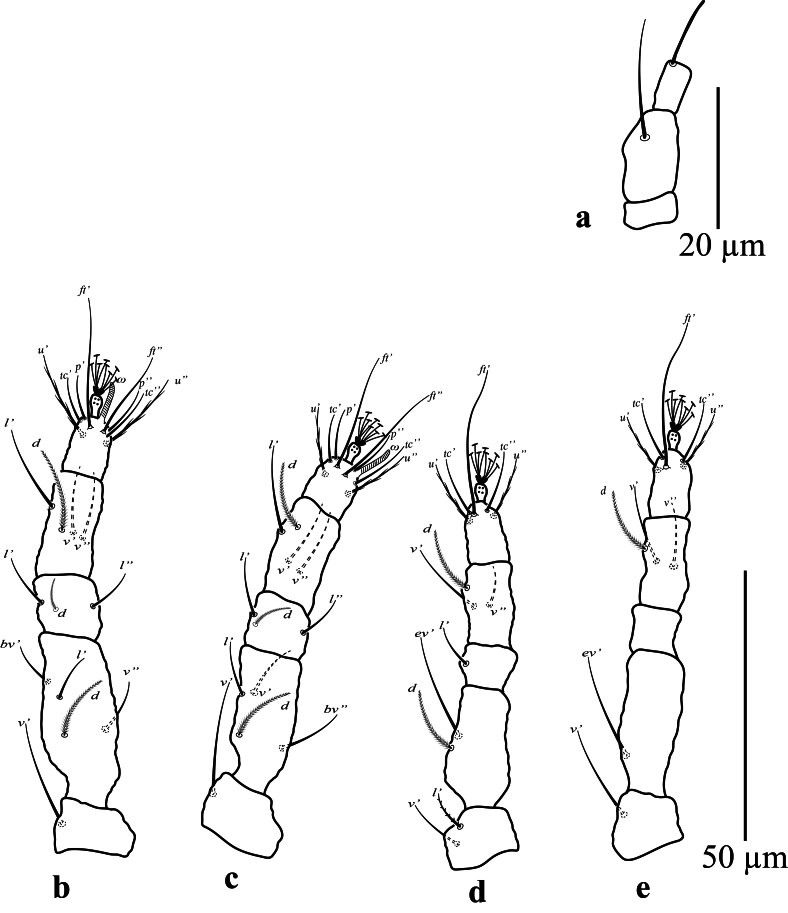
*Amblypalpuspisoniae* sp. nov., female; **a** palp; **b** leg I; **c** leg II; **d** leg III; **e** leg IV.

**Gnathosoma.** Ventral setae *m* 11 (10); distance between setae *m*-*m* 10 (13). Palp (Fig. 3a). Three segments; terminal segment with single eupathidium setae 10 (9), second segment with one dorsal seta.

**Legs** (Fig. 3b-e): Legs measured from trochanter to tarsus; leg I 84 (87); leg II 78 (75; leg III 72 (69) and leg IV 85 (78); Number of setae on leg I-IV (from coxa to tarsus, solenidion in parenthesis). Setal formula for legs I-IV: coxae 3–2–1–1; trochanters 1–1–2–1; femora 4–4–2–1; genua 3–3–1–0; tibiae 4–4–3–3; tarsi 8(1)–8(1)–5–5. Tarsal setae are unusually elongate, especially the unguinal setae *u′–u*″, companion setae *ft*″ and the proral eupathida *p′ζ–pζ*″. Tarsal claws pad-like.

**Description of male (n = 2)**

**Dorsum** (Fig. 4a, b). Propodosoma wider than opisthosoma. Length of body from setae *v*_2_-*h*_1_ 133–139; width from *sc*_2_-*sc*_2_ 75–77. Propodosoma anteriorly deeply notched 11–13, with two long medial lobes and without lateral lobes. Median area of propodosoma with irregular lines, lateral area with irregular longitudinal striae, lateral and sublateral area of propodosoma with irregular longitudinal striae; first and second pairs of propodosomal setae *v*_2_ and *sc*_1_ longer than third pair *sc*_2_. Opisthosoma with two plates; with irregular, longitudinal striae, opisthosoma with one pair of pore posterior setae *d*_3_. Lengths of setae: *v*_2_ 18–23, *sc*_1_ 25–26, *sc*_2_ 13–15, *c*_1_ 18–21, *c*_3_ 10–11, *d*_1_ 5–6, *d*_3_ 29–33, *e*_1_ 16–18, *f*_3_ 15–16, *h*_1_ 6–7, *h*_2_ 8–10. Distances between setae: *v*_2_–*v*_2_ 25–26, *sc*_1_–*sc*_1_ 49–50, *c*_1_–*c*_1_ 26–28, *c*_3_–*c*_3_ 80–82, *d*_1_–*d*_1_ 15–16, *d*_3_–*d*_3_ 62–65, *e*_1_–*e*_1_ 5–6, *f*_3_–*f*_3_ 44–46, *h*_1_–*h*_1_ 5–7, *h*_2_–*h*_2_ 26–28. All dorsal setae lanceolate serrate.

**Figure 5.**
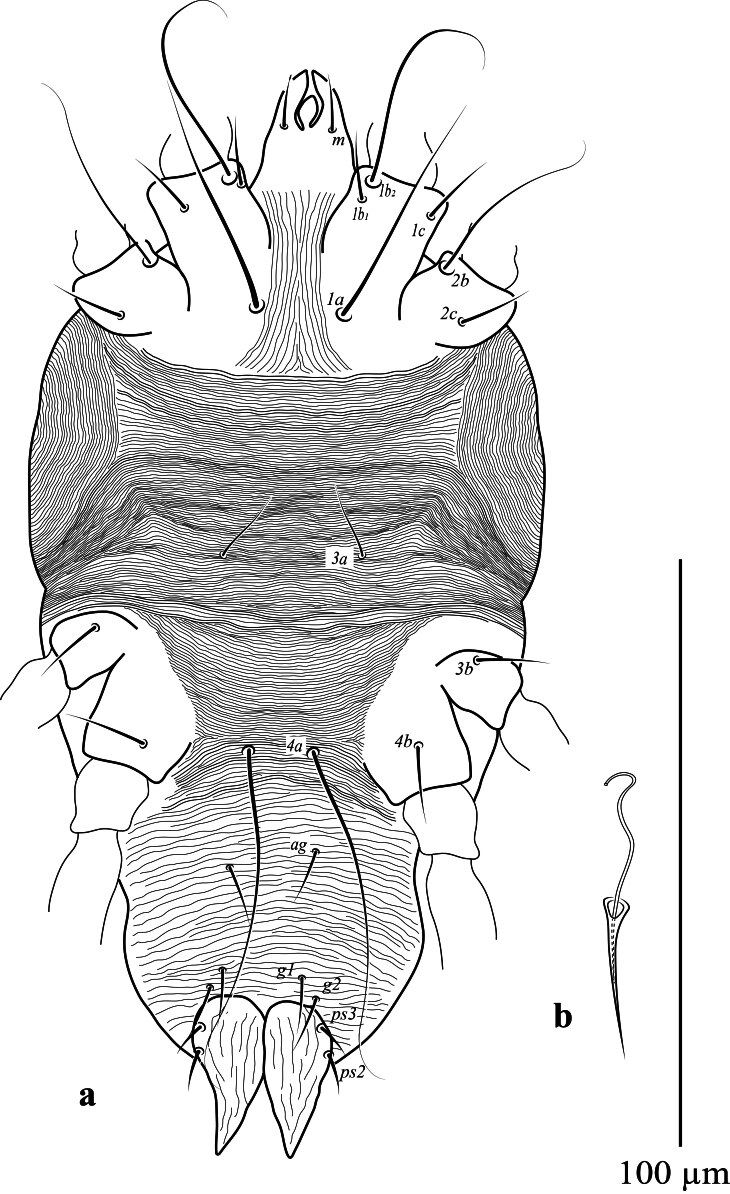
*Amblypalpuspisoniae* sp. nov., male. **a** venter; **b** aedeagus.

**Venter** (Fig. 5a). Ventral cuticle with transverse striae medially, longitudinal striae laterally; genital and ventral plate fused into single plate with transverse striae; lateral area with longitudinal striae; three pairs of intercoxal setae (1*a*, 3*a*, 4*a*), with 1*a* and 4*a* longer than 3*a*; Coxa I with 3 setae (1*b*_1_, 1*b*_2,_ 1*c*); two pairs of genital setae (*g*_1–2_) present; two pairs of anal setae (*ps*_2–3_) present. Lengths of setae: 1*a* 41–42, 1*b*_1_ 13–15, 1*b*_2_ 33–34, 1*c* 11–14, 2*b* 33–40, 2*c* 13–16, 3*a* 11–13, 3*b* 13–15, 4*a* 60–63, 4*b* 14–15, *ag* 10 10–11, *g*_1_ 9–11, *g*_2_ 10–11, *ps*_2_ 7–9, *ps*_3_ 7–11. Ventral setae smooth. Aedeagus as figured (Fig. 5b).

**Figure 6.**
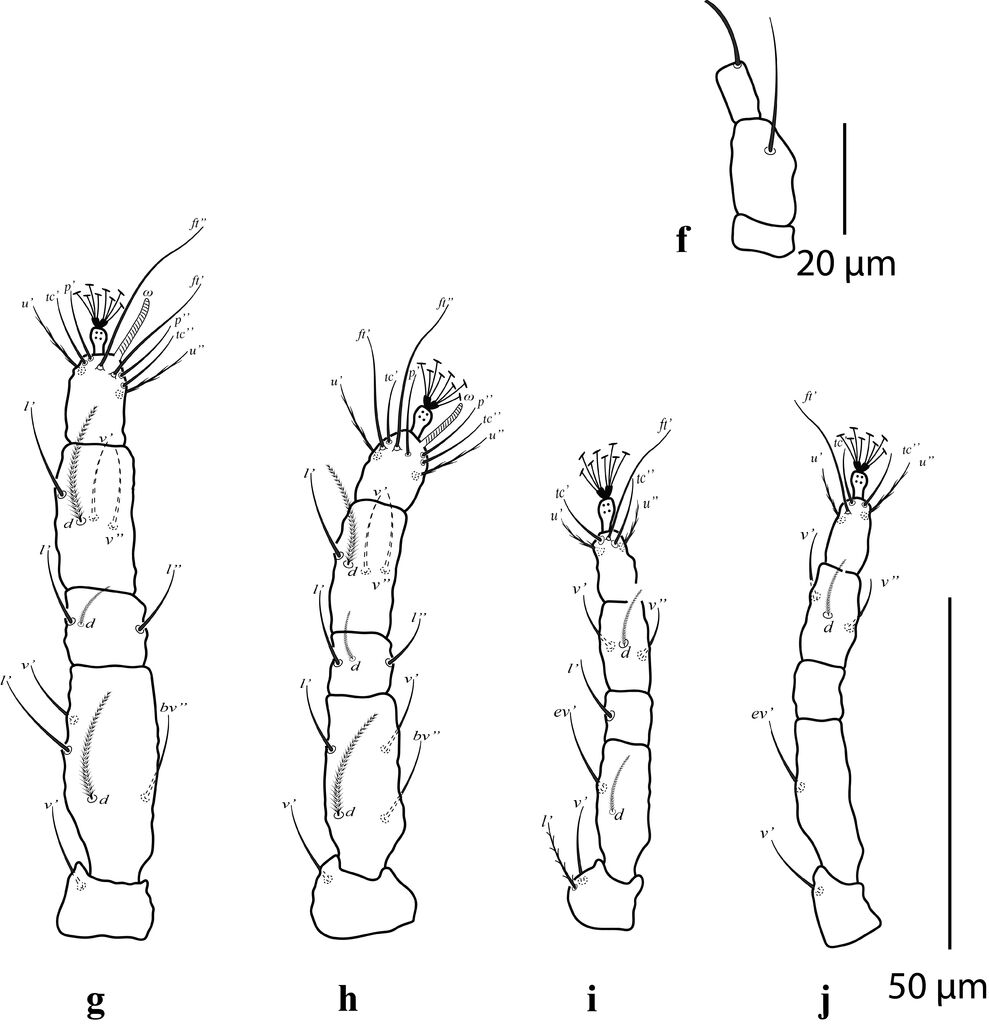
*Amblypalpuspisoniae* sp. nov., male. **f** palp; **g** leg I; **h** leg II; **i** leg III; **j** leg IV.

**Gnathosoma.** Ventral setae *m* 10 (10–12); distance between setae *m*–*m* 12–13. Palp (Fig. 6f). Three segments; terminal segment with single eupathidium setae 7–12, second segment with one dorsal seta.

**Legs** (Fig. 6g-j): Legs measured from trochanter to tarsus; leg I 84 (79–84); leg II 69 (69–74); leg III 66 (66–69) and leg IV 77 (77–80). Number of setae on leg I-IV similar to female legs.

**Immature stages.** Not found

##### Etymology

The specific epithet (*pisoniae*) is derived from the host plant genus *Pisonia* from which the type specimen was collected.

##### Genus diagnosis

**Type species**: *Amblypalpusnarsikulovi* Mitrofanov & Strunkova, 1978 - Mitrofanov and Strunkova 1978: 1097.

**Updated diagnosis** after Farzan et al. (2013)

Palps 3-segmented; palp tarsus with 1–2 terminal phaneres; anterior margin of prodorsum with broad flat projection extending medially and laterally over base of coxa I or projection absent; opisthosoma with 7–9 pairs of setae (*c*_2_, *d*_2_, *e*_2_, *f*_2_, absent; *c*_1_, *e*_3_, *f*_3_ present or absent); dorsal setae fine; leg cuticle not heavily sculptured; genital and ventral shield weakly developed or not at all defined; metapodal shields absent; two pairs of pseudoanal setae (*ps*_2–3_), anal plate poorly defined, setae *ps*_2_ not on tubercle, *ps*_2–3_ inserted longitudinally on anal plates; tibiae I–II with 4 or 5 setae; tarsal claws and empodia pad-like. Spermatheca with at least one subterminal bulb.

#### Diagnosis

##### Diagnosis

Propodosoma with anterior median projection deeply notched, rostral shield present; opisthosma with setae *c*_1_, *c*_3_, *d*_1_, *d*_3_, *e*_1_, *f*_3_, *h*_1_ and *h*_2_ present and setae *e*_3_ and *f*_2_ absent, all dorsal setae fine, serrate, moderately short, with *v*_2_, *sc*_1_, *c*_1_ the longest; dorsal idiosoma with irregular mostly fine striation; genital and ventral region fused with fine transverse striae; genu III with one seta; tibiae I and II with four setae; palp three segmented, distal segment with single eupathidium.

#### Distribution

##### Remarks

The new species belongs to *Amblypalpus* sensu lato. The new species *Amblypalpuspisoniae* sp. nov. is closely related to *A.iraniensis* Farzan, Asadi & Ueckerman, 2013 because both have palp with three segments, dorsum striated; genua I and II, femora I and II each with three and four setae, respectively. However, the new species can be distinguished from *A.iraniensis* by seta *e*_3_ absent (vs. seta *e*_3_ present); anterior medial propodosoma projection deeply notched (vs. anterior medial propodosoma projection reduced); seta *c*_1_ short, not reaching basis of seta *d*_1_ (vs. *c*_1_ crossing far away basis of seta *d*_1_); genito-ventral plate striated (vs. genito-ventral plate smooth); genu III with one seta (vs. genu III without seta) tibiae I and II, each with four setae (vs. tibiae I and II, each with five setae in *A.iraniensis*).

## Supplementary Material

XML Treatment for Amblypalpus pisoniae

## Figures and Tables

**Figure 1a.**
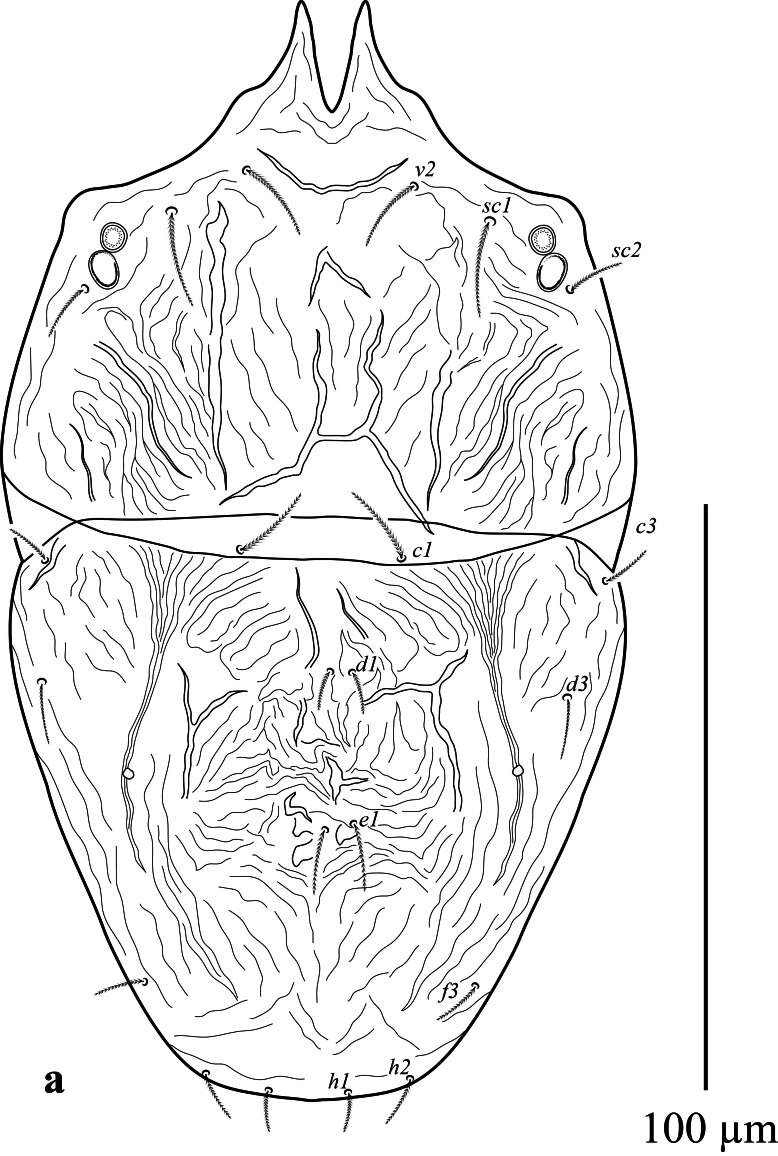
*Amblypalpuspisoniae* sp. nov., female, dorsum (Illustration);

**Figure 1b.**
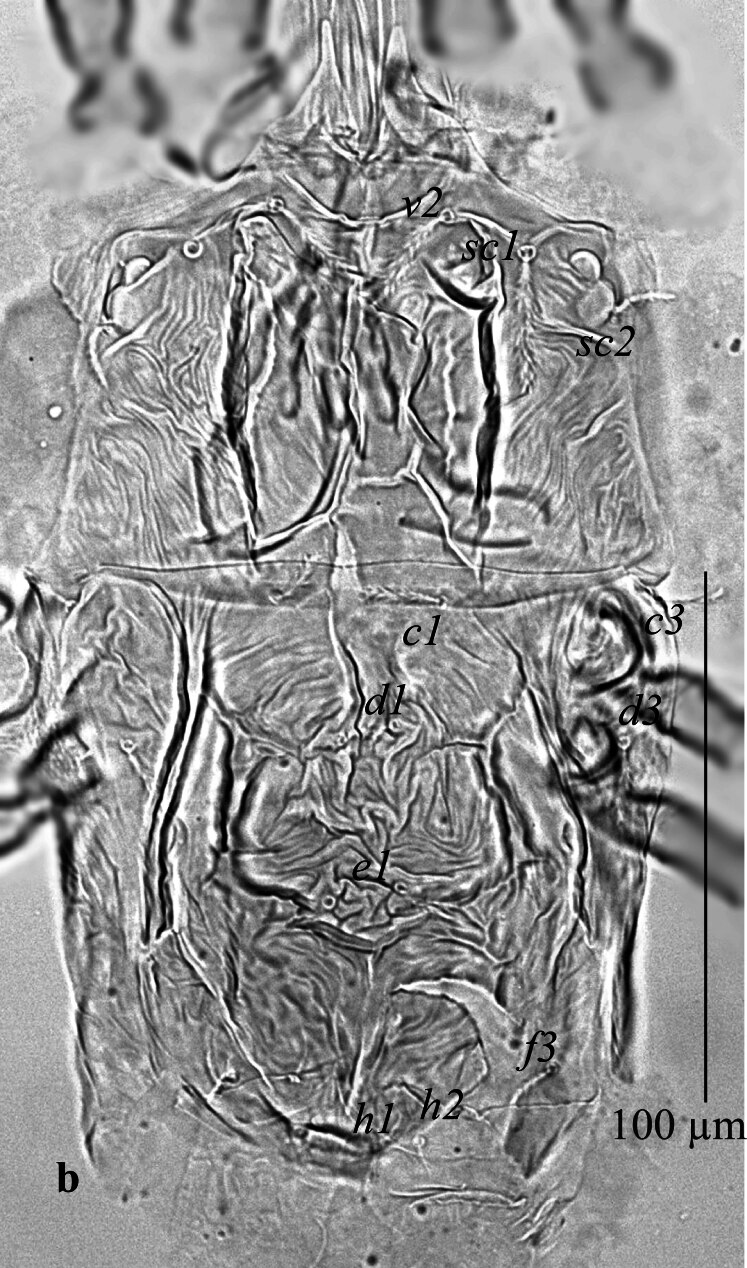
*Amblypalpuspisoniae* sp. nov., female, dorsum (Photo).

**Figure 2a.**
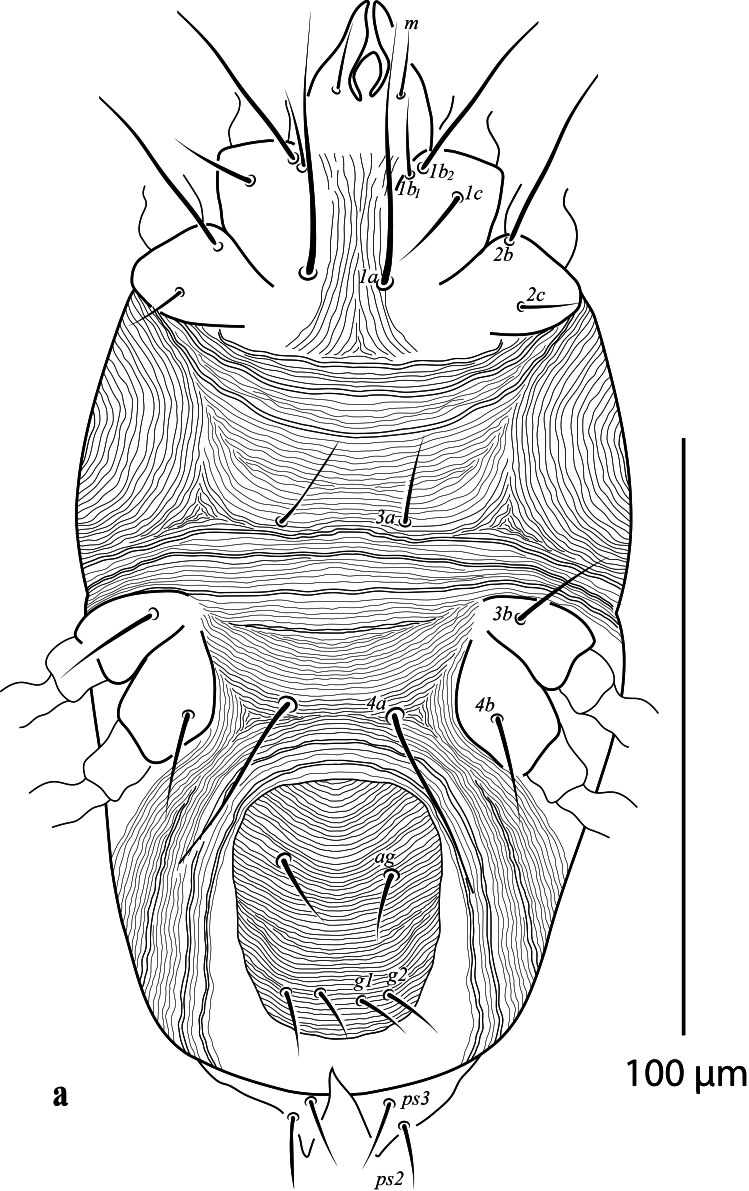
*Amblypalpuspisoniae* sp. nov., female, venter (Illustration);

**Figure 2b.**
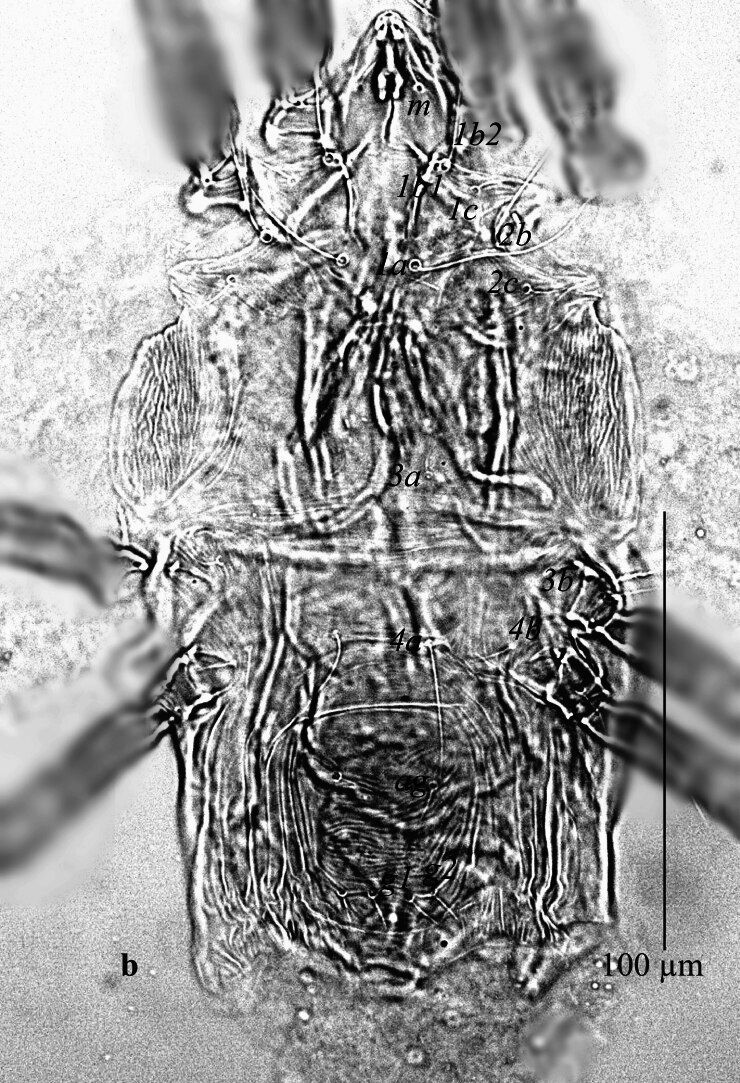
*Amblypalpuspisoniae* sp. nov., female, venter (Photo);

**Figure 2c.**
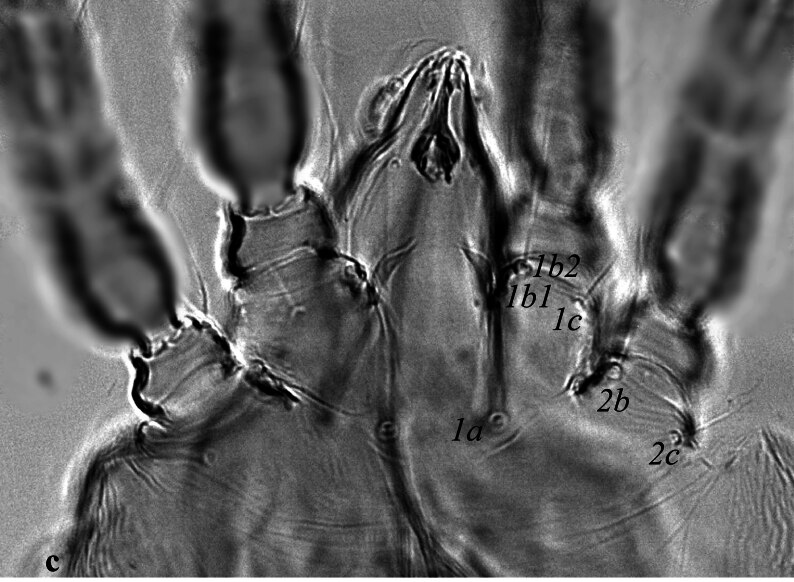
*Amblypalpuspisoniae* sp. nov., female, coxae I and II (Photo);

**Figure 2d.**
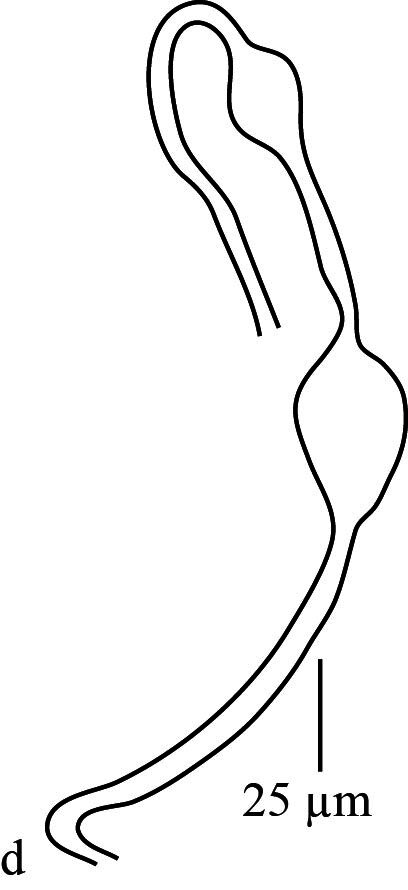
*Amblypalpuspisoniae* sp. nov., female, spermatheca (Illustration).

**Figure 4a.**
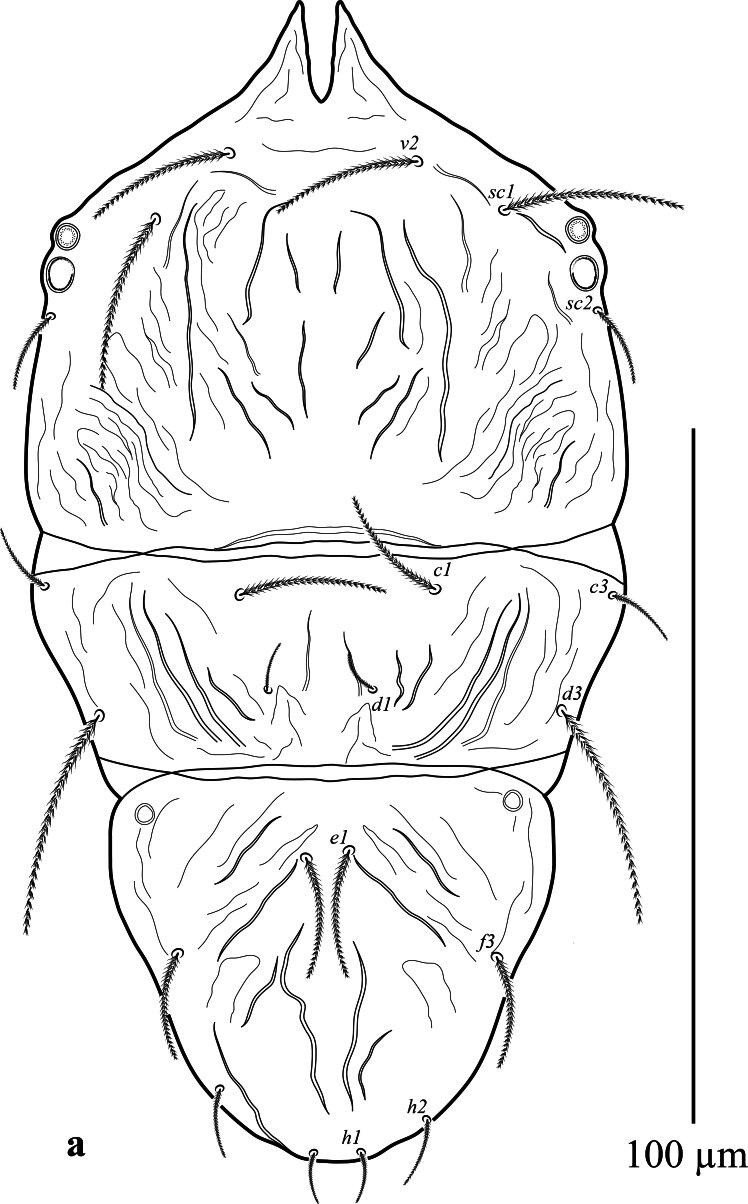
*Amblypalpuspisoniae* sp. nov., male, dorsum (Illustration);

**Figure 4b.**
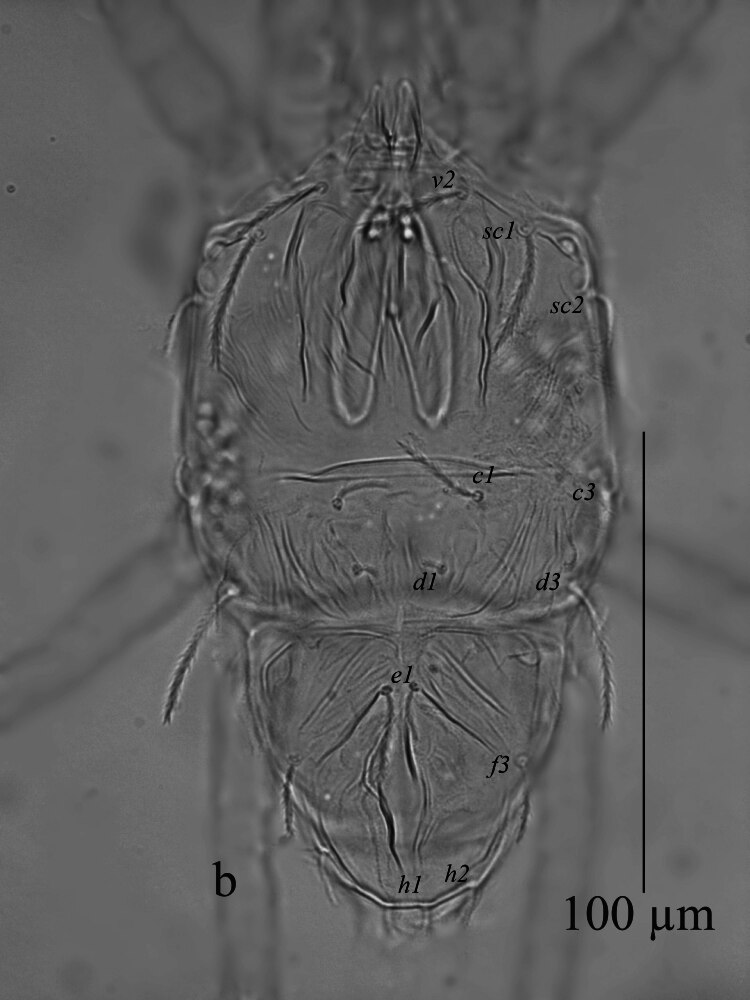
*Amblypalpuspisoniae* sp. nov., male, dorsum (Photo).
